# Purposes of Smartphone Health Care Apps and the Practicality of Their Functions in Disaster Situations: Qualitative Function Assessment Study

**DOI:** 10.2196/56862

**Published:** 2025-03-05

**Authors:** Sayuri Nonaka, Susumu Fujii, Kosuke Chris Yamada

**Affiliations:** 1 Disaster Medical Informatics Lab, International Research Institute of Disaster Science, Tohoku University, Sendai, Japan; 2 Research Institute of Sport Medical Science, Tokai University, Hiratsuka, Japan

**Keywords:** health care, disaster medicine, mobile app, survey, disaster situations, self-reliance, Japan, disaster response, mobile phone

## Abstract

**Background:**

Japan has experienced various natural disasters, including the Great East Japan Earthquake in 2011. It becomes crucial to focus on strengthening self-help measures through health care apps that are used in normal times to help people during disasters. However, little is known about what health care apps would be useful in times of disaster.

**Objective:**

This study aimed to investigate the prevalent functionalities and purposes of using health care apps during normalcy, explore their potential utility, and propose strategies for disaster response through their utilization.

**Methods:**

We focus on highly ranked health care apps (within the top 100 in the health care category for iPhones by Apple, Inc for a certain period) and reclassify their purpose of use, such as sleep, relaxation, and exercise, in detail. We also investigate the functions within each health care app (measurement, recording, advice, content provision, and guidance to actual services), based on which we determine their potential utilization during disasters and anticipate potential solutions to address disaster-related challenges. We also consider the ideal framework of health care apps in disaster response, exploring possibilities such as the necessity of new disaster-specific apps or the adaptation of existing health care apps for disaster scenarios.

**Results:**

Among the 70 free apps, the predominant functions included “recording” (n=60 cases, 86%) and “measurement” (n=47 cases, 67%), primarily encompassing the mechanical functions of wearable devices and smartphones. A similar trend was seen in the 77 paid apps, but “content provision” (n=54 cases, 70%) was the most prevalent. Furthermore, the “content provision” function was particularly common in the “purposes of use” categories “sleep” (*χ*^2^_11_=29; *P*<.001), “relaxation” (*χ*^2^_11_=14.6; *P*<.001), and “exercise” (*χ*^2^_11_=9.3; *P*=.002). This suggested the possibility of using the content provision function in existing health care apps to support mental and physical health even during a disaster.

**Conclusions:**

The widespread use of apps during normal times could minimize hesitation in adopting them during disasters. The findings emphasize the potential for augmenting disaster-specific content within existing apps rather than developing new ones. This approach aligns with the likelihood of preinstalled app use during emergencies, indicating a pragmatic strategy for enhancing disaster response content within prevalent apps.

## Introduction

Japan has experienced various natural disasters, including the Great East Japan Earthquake (GEJE) in 2011 [1-3], the Kumamoto earthquake in 2016, and wind and flood damage induced by Typhoons 15 and 19 in 2019 [4-6]. Furthermore, there is a 70% probability of a Nankai Trough earthquake and an earthquake directly under the Tokyo metropolitan area by 2050, necessitating urgent attention to disaster prevention, mitigation, and response [7].

The issues in drug prescribing that became apparent after the GEJE have been partially resolved using the Medication Record Book held by those affected by the disaster [8]. Electronic standards such as E-Kusu links have been established, and many drugstores and local governments are transitioning from paper-based to electronic medication handbooks [9]. Furthermore, various issues, such as managing chronic diseases, including diabetes and mental health during disasters, have been identified [10-17]. To reduce the burden on public and mutual aid support systems in times of disaster, we thought that familiar digital technology, health care apps for smartphones, could be expected to strengthen self-help [18-21]. In this study, we focused on strengthening self-help measures during disasters using health care apps that are used in everyday life.

The best way to determine whether an app can be used effectively during a disaster is to investigate whether it can be used during a disaster. However, it is difficult to conduct such a survey during a disaster. In addition, it is time-consuming to apply the findings to the next disaster. Therefore, we assume that apps commonly used in the health care field related to human life will be immediately available at the time of a disaster and divide them into subgenres related to the issues at the time of a disaster. The potential for use in times of disaster was considered by surveying the implemented IT functions. This is because we thought that it would be difficult to use during a disaster if the function is mainly just measurement and record keeping. If the function can provide detailed methods such as content, there is a high possibility that it can be used during a disaster. In other words, rather than creating an app that provides content useful only for disasters, we thought it would be more effective to add only content for disaster apps within an app already installed and tied to the issues at the time of a disaster.

This study aimed to investigate the prevalent functionalities and purposes of using health care apps during normalcy, explore their potential utility, and propose strategies for disaster response through their utilization.

Specifically, we focus on highly ranked health care apps (within the top 100 in the health care category for iPhones by Apple, Inc for a certain period) and reclassify their purpose of use, such as sleep, relaxation, and exercise, in detail. We also investigate the functions within each health care app (measurement, recording, advice, content provision, and guidance to actual services), based on which we determine their potential utilization during disasters and anticipate potential solutions to address disaster-related challenges. We also consider the ideal framework of health care apps in disaster response, exploring possibilities such as the necessity of new disaster-specific apps or the adaptation of existing health care apps for disaster scenarios.

## Methods

### Survey Procedure

#### Overview

In this study, we conducted a comprehensive survey of current health care–related smartphone apps to determine their functionalities and intended purposes. Subsequently, we investigated the functions implemented for each specific purpose. Finally, based on the findings, we evaluated the potential utilization of each purpose during disaster scenarios, identifying their utility and challenges.

The procedure that we followed for the surveys is elaborated below.

#### Initial Selection (Survey 1)

For approximately 3 months, we targeted commonly used daily life health care or fitness apps by many Japanese people that were ranked within the top 100 on app download sites. The download site is Apple’s App Store.

#### Functional Survey (Survey 2)

We focused on technical functionalities such as measurement (including wearable device integration), data recording, advice, and video provision. We classified functionalities into 5 categories: measurement function, recording function, advice provision function, content provision function, and actual service function.

#### Purpose Classification (Survey 3)

We surveyed and categorized the intended purposes for each app into eating habits, self-management, sleep, relaxation, walking, running, exercise, cycle management (menstruation or pregnancy), habit formation, childcare or parenting, teaching materials or practice, and actual service provision.

#### Analysis of Characteristics of Apps (Survey 4)

We examined the characteristics of functional implementation unique to each purpose identified in surveys 2 and 3. We also considered how to use them in the event of a disaster.

### Survey Description

#### Overview

Using the cumulative results from surveys 1 to 4, we assessed the utility and challenges of each purpose during disaster situations. We evaluated whether the existing system could be directly used or required customization to address disaster-specific issues. Detailed descriptions of each survey method follow below.

#### Survey 1: Targeted Apps (Commonly Used Daily-Life Health Care Apps)

We included iPhone apps available on the App Store of Apple, Inc. There was no category for emergencies or disasters in the App Store, so the category for medical or health care–related options would be the best choice for dealing with disasters. According to the App Store category descriptions, the “Medical” category is described as “Apps that are focused on medical education, information management, or health reference for patients or health care professionals” [22]. The “Health & Fitness” category comprises “Apps related to healthy living, including stress management, fitness, and recreational activities” [22]. We targeted apps in the “Health & Fitness” category because they are accessible for general daily use rather than exclusively for health care professionals. The survey period was from October 2021 to April 2022, and the data were collected 4 times. The target apps were chargeable and free in the “Health & Fitness” category displayed exclusively on iPhones, excluding rankings on iPads and Macs. In addition, all apps had to be ranked within the top 100 during all 4 data collection periods. The App Store has a primary category for the app. If the category is inappropriate for an app, it violates the guidelines [22]. Whether an app is classified in the “Health & Fitness” category is determined by Apple, Inc.

#### Survey 2: Classification of “Implemented Function”

This survey aimed to identify 5 distinct functions (A: measurement function, B: recording function, C: advice provision function, D: content provision function, and E: actual service function) within the health care apps targeted in survey 1. Assessment of function implementation relied on scrutinizing app descriptions and screenshot images available in the apps. We did not verify these functions’ actual performance or effectiveness or whether they precisely aligned with the description provided. As there is no existing classification system for these 5 functions, researchers (SF and KCY) who are an expert in app development and evaluation defined them, and another expert (SN) carried out the classification work. Multiple researchers discussed any doubt about the decision. The elucidation of each function is as follows.

- “A: measurement function” was examined to determine whether the function displays numerical values (measured values) by linking with smartphone sensors or external devices worn by individuals (eg, wearable devices and home-use measuring devices). Information on whether the values were stored was not sought. If the description mentioned support for the Apple health care app or Health Kit, it was deemed linked to a wearable device and was considered to possess a “measurement” function. If the value could only be entered manually, no “measurement function” was provided.
- “B: recording function” was defined as the ability to link, read, and save (record) values acquired from manual inputs and view the data. Information on the storage duration (history period) or storage frequency (history count) was sought. The recorded values could be manually entered, read from external data, or include sensor values obtained from “A: measurement function.”
- “C: advice provision function” focused on investigating whether the app offered advice to users from various sources, including dedicated communities, experts, or artificial intelligence functions. The advice content was not limited to advice optimized based on personal data, advice from nonexperts in the community, or generalized advice. However, supportive advice, such as encouraging users, was not considered. Additionally, advice that only explained how to use the app or explained the provided content was not considered.
- “D: content provision function” was defined as providing specific health care methods (content), such as videos and recipes, within the app.
- “E: actual service function” focused on scrutinizing whether health care services were provided through human intervention. Services were assumed to be provided through digital services, such as scheduling appointments at physical stores, phone calls, and chats.

**Figure 1.**
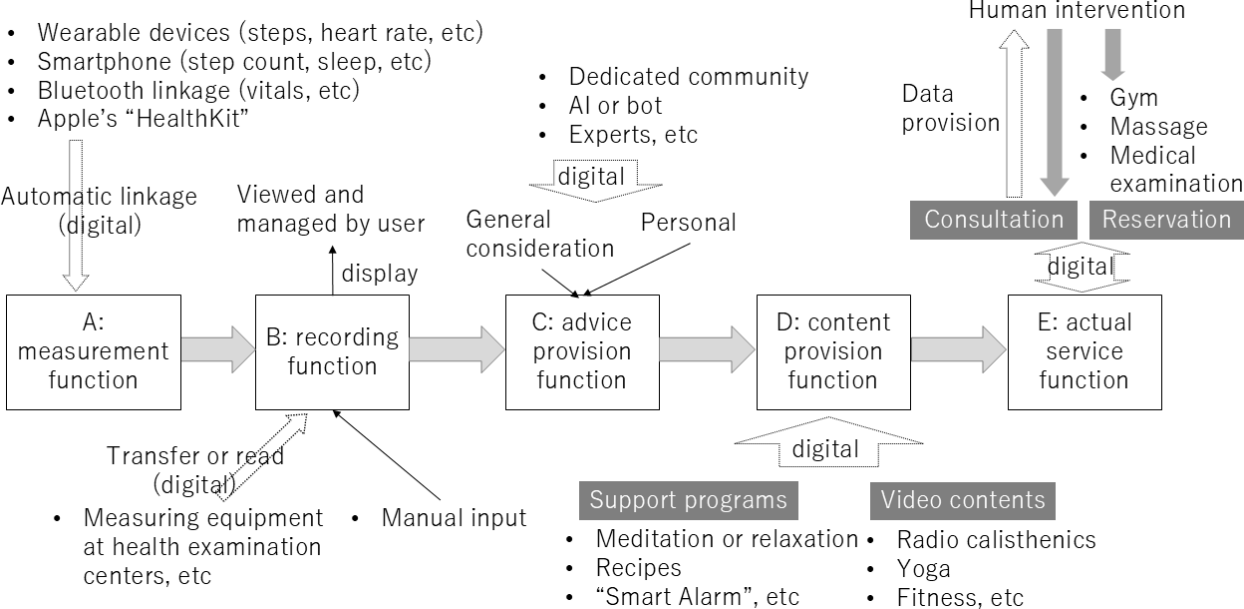
Classification of functions. The function of an app through a series of flow diagrams, depicted as a sequence from A to E. Data measured by the user is recorded, advice is offered based on the recordings, content aligned with the advice is provided, and the actual service is offered in the form of human intervention. AI: artificial intelligence.

Figure 1 illustrates an app’s function through a series of flow diagrams, depicted as a sequence from A to E.

#### Survey 3: Categorization of “Purpose of Use”

Survey 1 results, categorized broadly under the health care classification by Apple, were reclassified into more detailed purposes of use because of the initial classification’s broadness. The reclassification aimed to offer a more granular breakdown of the apps’ intended purposes and was based on the service purpose outlined by the provider without considering any unintended or user-applied purposes of use. Determining the purpose of use relied on the information provided within the app description and screenshot images. Initially, the apps were classified into 15 categories (Multimedia Appendix 1), including “eating habits,” “sleep,” “relaxation,” “walking,” “running,” “exercise,” “cycle management (menstruation or pregnancy),” and “childcare or parenting.” If an app did not fit any of these categories, they were further classified, and if an app served multiple purposes, it was assigned multiple purposes of use. As there is no existing classification system for this category, researchers (SF and KCY) who are an expert in app development and evaluation defined them, and another expert (SN) carried out the classification work. Multiple researchers discussed any doubt about the decision.

#### Survey 4: Evaluation of Cross-Tabulation Results Between “Implemented Function” and “Purpose of Use”

Survey 4 involved a cross-tabulation evaluation between the purpose of use (survey 3) and the implemented functions (survey 2) classifications. The primary objective was to explore any notable characteristics in the implemented functions (A to E) concerning each purpose of use. For instance, the assessment aimed to discern commonalities, such as whether “A: measurement function” and “B: recording function” were prevalent in apps designed for sleep-related use or whether “A: measurement function” was rare in apps offering actual services. We also analyzed which “implemented function” was prominently implemented for each “purpose of use.” Multiple response test was used for the analysis. A commercially available software program JMP (version 17.1.0; SAS Institute) was used for the analyses. This survey was compiled and analyzed by an expert health care app specialist (SN) and verified by some experts (SF and KCY) in disaster medicine. This evaluation, encompassing surveys 1‐4, facilitated discussions on the utility and challenges posed during disasters for each purpose of use. Additionally, it provided insights into precautions and suggestions for customization to enhance utility during disaster scenarios.

### Ethical Considerations

This study does not involve the collection of data or interventions with human participants but is based on existing published app rankings and app descriptions. Therefore, we confirmed that this study did not meet the ethical review requirements at our institution. More specifically, this study satisfied the following conditions: it did not involve experiments, observations, or interventions in the human body. It did not collect or reuse personal information from participants and used only publicly available information, so the identification of individuals is impossible.

## Results

### Result 1: Targeted Survey of Commonly Used Daily-Life Health Care Apps

Apps in Apple’s “Health & Fitness” category included those with “in-app purchases” (Apple Inc, 2023) for both free and paid downloads. “In-app purchases” included “consumable,” “nonconsumable,” “auto-renewable subscriptions,” and “nonrenewing subscriptions” [23]. The presence of “in-app purchases” was not used to distinguish between free and paid download apps and those that required a fee to commence use were classified as paid download apps; Apple’s classification at the time of the survey was used. The evaluation of free apps occurred on 4 occasions: October 22, 2021; October 29, 2021; November 22, 2021; and January 11, 2022. Paid apps were similarly scrutinized on 4 different dates: February 2, 2022; February 22, 2022; March 22, 2022; and April 12, 2022. In the second survey, the proportion of free apps was 86%, while that of paid apps was 84%. In the third survey, these values changed to 77% and 81%, respectively. By the fourth survey, the proportions further declined to 70% for free apps and 77% for paid apps. The results of survey 1 indicated that 147 (70 free and 77 paid) apps ranked in the top 100 on all 4 occasions.

### Result 2: Classification of “Implemented Function”

**Figure 2.**
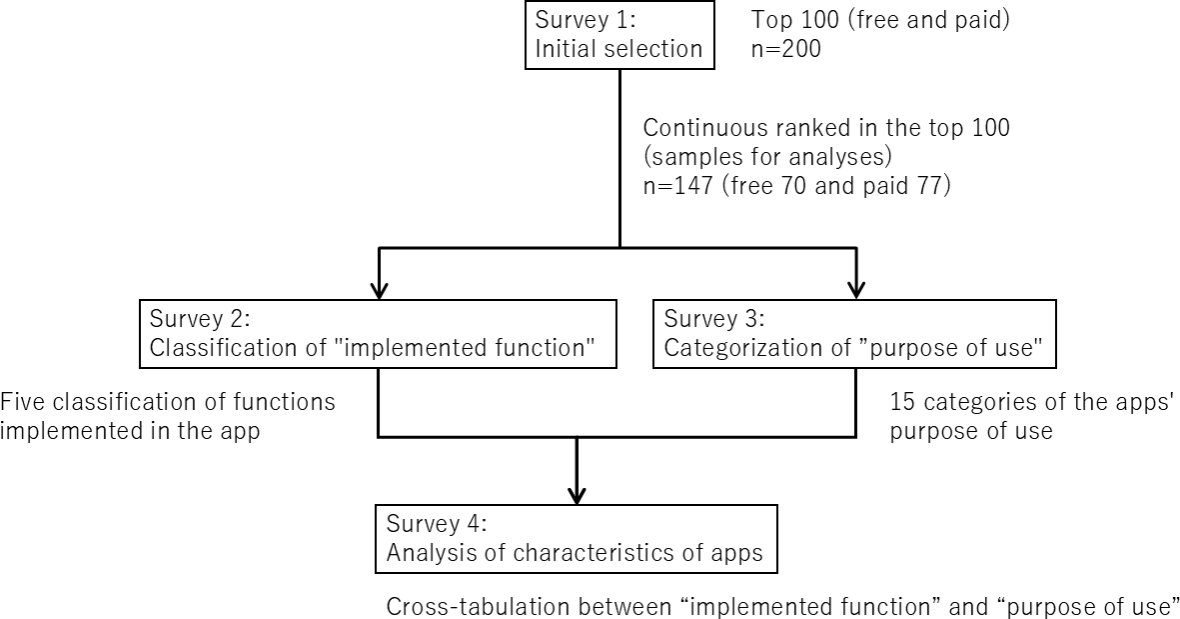
Survey procedure overview. After survey 1 narrowed the target apps to 147, we examined the functions implemented in these apps (survey 2) and the purpose of using these apps (survey 3). Survey 4 analyzed the characteristics of apps based on their implemented functions and purpose.

Figure 2 provides an overview of the survey procedure. The results regarding the implemented functions within the apps are summarized in Table 1. It includes the 147 apps from Result 1, which ranked in the top 100 across all 4 survey points. Among the 70 free apps, the predominant function was “B: recording function” (n=60 cases, 86%), followed by “A: measurement function” (n=47 cases, 67%). These functionalities primarily leveraged the mechanical capabilities inherent in wearable devices and smartphones. The 77 paid apps also displayed a high implementation rate for “B: recording function” (n=48 cases, 62%) and “A: measurement function” (n=39 cases, 51%); however, the most notable difference lay in the implementation of “D: content provision function” (n=54 cases, 70 %) within paid apps, showcasing a higher rate than in free apps (n=28 cases, 40%). The app vendor specifically curated this content, marking a distinct trend from the mechanically driven performance witnessed in free apps.

**Table 1.** Results of classification of functions implemented in the app.

	A: measurement	B: recording	C: advice provision	D: content provision	E: actual service
Free apps^a^ (n=70), n (%)	47 (67)	60 (86)	32 (46)	28 (40)	10 (14)
Paid apps^b^ (n=77), n (%)	39 (51)	48 (62)	18 (23)	54 (70)	0 (0)
Total downloaded apps (n=147), n (%)	86 (58.5)	108 (73.5)	50 (34)	82 (55.8)	10 (6.8)

a “Free” means free download apps.

b “Paid” means paid download apps.

### Result 3: Categorization of “Purpose of Use”

The results of the “purpose of use” categorization are summarized in Table 2. Apple’s categorization had a coarse granularity regarding the apps’ “purpose of use”; therefore, the recategorization was performed. No additions other than the 15 “purpose of use” categorizations set during planning were performed. Among the 70 free apps, “walking” was the most common (n=16 cases, 23%), followed by “exercise” and “self-management” (n=15 cases each, 21%). Among the 77 paid apps, “sleep” (n=21, 27%) and “relaxation” (n=20, 26%) were the most common, followed by “exercise” (n=15, 20%). Overall (147 combined free and paid apps), the most popular activities were “exercise” and “sleep” (n=30 cases each, 20.4%), followed by “relaxation” (n=28, 19%) and “self-management” (n=25, 17%). Note that only paid apps were available for “childcare or parenting” and “teaching materials or practice,” whereas only free apps were available for “actual service provision.” The designation “not applicable” refers to apps lacking health care functionality. For the categories of “self-management,” “habit formation,” “teaching materials or practice,” and “tools for Apple Health,” a single purpose of use was assigned if it could not be determined to be the primary purpose of use, even if multiple purposes of use were plausible. However, if an app implied multiple purposes, such as “eating habits” and “exercise,” the apps were tallied separately within each applicable category, leading to a cumulative count across various purposes.

**Table 2.** Results of the categorization of the apps’ purpose of use.

Purpose of use	Free (n=70), n (%)	Paid (n=77), n (%)	Total (n=147), n (%)
Eating habits	10 (14)	3 (4)	13 (8.8)
Sleep	9 (13)	21 (27)	30 (20.4)
Relaxation	8 (11)	20 (26)	28 (19)
Walking	16 (23)	2 (3)	18 (12.2)
Running	5 (7)	2 (3)	7 (4.8)
Exercise	15 (21)	15 (20)	30 (20.4)
Cycle management (menstruation or pregnancy)	8 (11)	4 (5)	12 (8.2)
Childcare or parenting	0 (0)	3 (4)	3 (2)
Actual service provision	10 (14)	0 (0)	10 (6.8)
Self-management	15 (21)	10 (13)	25 (17)
Habit formation	2 (3)	4 (5)	6 (4.1)
Teaching materials or practice	0 (0)	4 (5)	4 (2.7)
Tools for Apple Health	0 (0)	3 (4)	3 (2)
Not applicable	9 (13)	0 (0)	9 (6.1)
Pack of apps	0 (0)	2 (3)	2 (1.4)

### Result 4: Evaluation of Cross-Tabulation Results Between “Implemented Function” and “Purpose of Use”

The survey was conducted to determine the characteristics of the provided functions according to the purpose of use. “Tools for Apple Health,” “pack of apps,” and “not applicable” were excluded from survey 4 because they were not directly relevant to health-related purposes of use. The results of combining the “implemented function” categorized in survey 2 for each “purpose of use” classified in survey 3 are presented in Table 3.

**Table 3.** Cross-tabulation results of “implemented function” classifications by “purpose of use” of apps.

Purpose of use	A: measurement, n (%)	B: recording, n (%)	C: advice provision, n (%)	D: content provision, n (%)	E: actual service, n (%)
Eating habits (n=13)	12 (92)	13 (100)	10 (77)	11 (85)	1 (8)
Sleep (n=30)	17 (57)	16 (53)	11 (37)	30 (100)	1 (3)
Relaxation (n=28)	12 (43)	14 (50)	7 (25)	26 (93)	1 (4)
Walking (n=18)	18 (100)	18 (100)	8 (44)	8 (44)	2 (11)
Running (n=7)	7 (100)	7 (100)	5 (71)	6 (86)	0 (0)
Exercise (n=30)	21 (70)	27 (90)	13 (43)	26 (87)	2 (7)
Cycle management (menstruation or pregnancy; n=12)	8 (67)	12 (100)	11 (92)	7 (58)	3 (25)
Childcare or parenting (n=3)	1 (33)	1 (33)	1 (33)	3 (100)	0 (0)
Actual service provision (n=10)	4 (40)	9 (90)	6 (60)	6 (60)	10 (100)
Self-management (n=25)	22 (88)	25 (100)	9 (36)	3 (12)	0 (0)
Habit formation (n=6)	1 (17)	6 (100)	0 (0)	0 (0)	0 (0)
Teaching materials or practice (n=4)	0 (0)	0 (0)	0 (0)	4 (100)	0 (0)

Table 4 examines trends in the “purpose of use” for each “implemented function.” For “A: measuring function,” a significantly greater number of respondents reported “eating habits,” “walking,” “running,” and “self-management,” while fewer respondents reported “relaxation,” “habit formation,” and “teaching materials or practice.” In the case of “B: recording function,” “eating habits,” “walking,” “cycle management,” and “self-management” were significantly more common, whereas “sleep,” “relaxation,” and “teaching materials or practice” were significantly less common. Regarding “C: advice provision function,” significantly more instances were found in “eating habits” and “cycle management” whereas “habit formation” reported significantly fewer occurrences. For “D: content provision function,” significantly more instances were observed in “sleep,” “relaxation,” “exercise,” and “teaching materials or practice,” whereas “self-management” and “habit formation” reported significantly fewer occurrences. In the case of “E: actual service function,” significantly more instances were noted in “actual service provision,” whereas “self-management” reported significantly fewer occurrences.

**Table 4.** Results of multiple response test: “purpose of use” categorized by “implemented function” of apps.

	*χ*^2^ (*df*)	*P* value
A: measurement function		
Eating habits	5.7 (11)	.02
Sleep	0.7 (11)	.41
Relaxation	5.1 (11)	.02
Walking	16.1 (11)	<.001
Running	6.3 (11)	.01
Exercise	0.5 (11)	.48
Cycle management (menstruation or pregnancy)	0.0 (11)	.84
Childcare or parenting	1.2 (11)	.28
Actual service provision	2.4 (11)	.13
Self-management	7.5 (11)	.006
Habit formation	5.7 (11)	.02
Teaching materials or practice	8.2 (11)	.004
B: recording function		
Eating habits	5.4 (11)	.02
Sleep	12.0 (11)	<.001
Relaxation	13.8 (11)	<.001
Walking	7.5 (11)	.006
Running	2.9 (11)	.09
Exercise	1.8 (11)	.18
Cycle management (menstruation or pregnancy)	5.0 (11)	.03
Childcare or parenting	3.3 (11)	.07
Actual service provision	0.6 (11)	.44
Self-management	10.4 (11)	.001
Habit formation	2.5 (11)	.11
Teaching materials or practice	13.4 (11)	<.001
C: advice provision function		
Eating habits	8.4 (11)	.004
Sleep	0.0 (11)	.92
Relaxation	2.0 (11)	.16
Walking	0.4 (11)	.55
Running	3.3 (11)	.07
Exercise	0.4 (11)	.52
Cycle management (menstruation or pregnancy)	15.6 (11)	<.001
Childcare or parenting	0.0 (11)	.88
Actual service provision	2.1 (11)	.15
Self-management	0.0 (11)	.87
Habit formation	5.7 (11)	.02
Teaching materials or practice	3.8 (11)	.05
D: content provision function		
Eating habits	3.3 (11)	.07
Sleep	29.0 (11)	<.001
Relaxation	14.6 (11)	<.001
Walking	2.2 (11)	.14
Running	2.0 (11)	.16
Exercise	9.3 (11)	.002
Cycle management (menstruation or pregnancy)	0.1 (11)	.81
Childcare or parenting	2.9 (11)	.09
Actual service provision	0.0 (11)	.91
Self-management	26.7 (11)	<.001
Habit formation	11.5 (11)	<.001
Teaching materials or practice	3.9 (11)	.049
E: actual service function		
Eating habits	0.0 (11)	.98
Sleep	0.9 (11)	.33
Relaxation	0.8 (11)	.38
Walking	0.3 (11)	.59
Running	1.1 (11)	.30
Exercise	0.0 (11)	.86
Cycle management (menstruation or pregnancy)	3.4 (11)	.06
Childcare or parenting	0.5 (11)	.49
Actual service provision	51.8 (11)	<.001
Self-management	3.9 (11)	.048
Habit formation	0.9 (11)	.33
Teaching materials or practice	0.6 (11)	.43

## Discussion

### Principal Results

The results from surveys 1‐4 provided insights into the potential usefulness of health care apps in the event of a disaster and highlighted specific concerns that need to be addressed for each purpose of use. We also discussed precautions and customization suggestions for use during times of disaster.

Survey 1 indicated that apps with enduring high rankings are more likely to be used during regular times. Considering the challenges of discovering and adopting new apps during disasters, using established apps beforehand could offer substantial advantages.

Survey 2 revealed that free apps primarily focus on measurement and recording, raising doubts about their direct applicability in times of disaster. By contrast, many paid apps offer content that can provide concrete advice, suggesting they could be used during disasters.

Survey 3 suggested a possibility of comprehensively addressing care demands during disasters by categorizing “exercise” as “physical” to maintain physical health and “sleep” and “relaxation” as “mental” (wellness). The survey also suggested using the app to provide comprehensive care during disasters.

Survey 4 explored the relationship between implemented functions and purpose of use, suggesting that health care apps require implementing functions such as recording and content provision. The implementation rate of content-providing functions was high for “physical” (eg, “exercise”) and “mental wellness” (eg, “sleep” and “relaxation”). In other words, apps frequently used during normal times may provide comprehensive (physical and mental) and specific content (response methods) during disasters. Using them during disaster scenarios is highly beneficial as they are already installed and configured.

In the following subsections, we discuss the anticipated effects of the apps’ specific “purpose of use” during disasters and suggest ways to improve their effectiveness in such scenarios, considering the apps’ “implemented functions.” The timing of using health care apps after a disaster is thought to vary from person to person. Therefore, the disaster phase we will examine here is the period after evacuation and after securing personal safety.

### “Eating Habits” Apps

Eating habits during disasters are biased toward carbohydrates such as rice, bread, and noodles, rendering maintaining a nutritionally balanced diet difficult [24]. Moreover, this can lead to life-threatening problems for those with allergies or chronic illnesses because they cannot prepare their meals [25]. A study reported that, following the GEJE, patients with type 1 diabetes required 6 months for their HbA_1c_ values to return to predisaster levels [26].

The “eating habits” apps are characterized by their “C: advice provision function”; however, solely relying on this function in situations such as evacuation shelters can be challenging. In such times, obtaining suitable food items and meals customized to individual situations becomes crucial but can also be difficult to procure. For instance, devising a meal tailored to one’s circumstances, especially considering evacuation rations, poses a significant challenge. Future endeavors should implement the “D: content provision function” to provide specific content. Moreover, the “C: advice provision function” includes a feature that allows information to be exchanged within a dedicated community. This feature enables the people impacted by the disaster to mutually exchange information and receive advice from medical specialists and nutrition managers.

### “Sleep” and “Relaxation” Apps

Daily sleep is an indicator of mental health [27]. Based on sleep status, even in the event of a disaster, the early detection of mental health problems may be possible [28,29].

Many of the “sleep” and “relaxation” apps were equipped with “D: content provision function.” Several users used paid apps, indicating that they have routine difficulties with “sleep” and “relaxation” and seek content that addresses these concerns. Although whether the content has a scientific basis is debatable, sleep and relaxation are particularly important for maintaining mental health even in times of disaster, and the possibility of providing a concrete method (content) can render the content useful in disaster situations. In the future, providing content tailored to the special environment of a disaster, such as living in an evacuation center, will be crucial.

### “Walking,” “Running,” and “Exercise” Apps

In evacuation centers, space limitations restrict physical activity, leading to a sedentary lifestyle and potential health issues such as poor circulation and muscle weakness due to a lack of exercise. Physical exercises, particularly for older adults, play a crucial role in preventing frailty [30]. However, challenges were observed during the GEJE, where exercise acceptance within evacuation centers was limited [31]. To address these issues, strategies have been proposed to include exercise instructors in disaster medical assistance teams and form a disaster-exercise support network, emphasizing collaboration with other organizations and exercise initiatives that include local governments [32].

While “walking” and “running” apps showed no significant implemented functions, the “exercise” apps tended to have more “D: content provision function.” Notably, “A: measurement function” was significantly implemented in “walking” and “running” apps. However, measuring the number of steps or distances might offer limited utility during disasters. Unless a specific means of “exercise” is provided, effectively using the system in the event of a disaster will be difficult. The challenge will be to provide an “exercise” content that can be implemented within limited spaces, including evacuation centers.

### “Cycle Management (Menstruation or Pregnancy)” Apps

In the aftermath of the GEJE, female evacuees encountered challenges, including inadequate sanitary products, underwear, and essentials for infants and young children [33,34]. Women (especially pregnant women and caregivers) are at higher risk of facing mental health problems during disasters [35,36], highlighting the stressful environment for several women in such situations. Frameworks such as The Sendai Framework for Disaster Risk Reduction, which describes women’s perspectives and participation in disaster reduction and building women’s capacities [37], and guidelines from the Cabinet Office (“Women’s Perspective for Strengthening Disaster Response Capabilities: Disaster Prevention and Recovery Guidelines from a Gender Equality Perspective”) [38,39] focus on enhancing women’s roles in disaster reduction and recovery.

Several women manage their menstrual and pregnancy cycles during normal times, suggesting that incorporating health care apps into their daily lives might be straightforward. For instance, as of 2022, the “LunaLuna” app, one of the “cycle management” apps, has been downloaded more than 19 million times [40].

By predicting these things, we can predict the demand for sanitary products, determine what is in short supply, and know when there is a problem with our bodies, such as when the cycle is different from normal. We can use this app effectively.

### “Childcare or Parenting” Apps

Only 3 apps were identified, all of which were paid apps used by parents to support their children’s development and growth. Therefore, their use during disasters would be challenging.

### “Actual Service Provision” Apps

The “actual service” apps provided convenience to existing members, such as digital reservations and consultations. Currently, these apps are only intended for use during normal times. However, they could prove useful during evacuations if they offer real services related to supplies and medical care.

### “Self-Management” Apps

Previous studies have demonstrated the potential benefits of using wearable devices to track activity, physiological functions, and lifestyle data. Having undergone rigorous testing for data accuracy, they can have a positive physical and mental impact [41-44].

The “self-management” apps tended to implement “A: measurement function” and “B: recording function,” but lacked “C: advice provision function” and “D: content provision function.” Currently, these apps only measure and record data from wearable devices, with limited use of the collected data.

However, ongoing research on wearable device technology suggests that it may become an essential tool in future health care apps by facilitating behavioral change and offering benefits to health care professionals and patients [45]. To date, most “self-management” apps focus on measurement and recording, rendering them less effective during a disaster scenario.

### “Habit Formation” Apps

These apps primarily encouraged behavioral change through reminders and consistent recording. However, its utility during disaster situations seems improbable at present.

### “Teaching Materials or Practice” Apps

These apps served only “D: content provision function.” They could prove useful if tailored precisely to specific needs. Creating content based on accurate knowledge of the situation would be safer and more precise during disasters than a general internet search. They might serve as a straightforward contingency preparedness tool not typically used in normal circumstances.

### Overall Perspective

Disaster situations require health care apps that not only measure and record day-to-day activities but also provide specific methods and actual services addressing unique disaster-related issues. These apps should encompass functionalities that can provide concrete methods specially tailored to disaster situations. We believe this is feasible using the content provision function in this study. For example, in terms of food, the blood sugar levels of diabetics will likely be difficult to control when eating emergency food in the event of a disaster. Emergency food generally consists mainly of carbohydrates, so there is a risk of sudden fluctuations in blood sugar levels and high blood sugar. In addition, there is a possibility that dehydration symptoms will worsen diabetes symptoms due to a lack of hydration due to concerns about using the toilet [46]. Therefore, if the app content could encourage people to drink more water and provide information on the fact that the rice balls and sweet buns provided immediately after a disaster contain sodium and the recommended amount of food to eat, it could be very useful. However, avoiding hypoglycemia and replenishing sugar levels would still be necessary.

Similarly, there is a trend for apps that deal with issues such as sleep, exercise, and stress management during disasters to have a significant implementation of the “D: content provision function,” which also suggests the possibility of managing health in terms of both “mental” and “physical” aspects even during disasters. In other words, the key is not to develop new apps but to prepare content specific to disasters. Because these apps are widely used normally, they are highly reliable and have low initial implementation costs. Therefore, enhancing existing content is more reasonable than developing new apps as disaster countermeasures.

### Limitations

There are some limitations to this study. First, this study focused only on Apple’s App Store apps. Although we assumed that the top 100 apps were used among all the users of smartphones, they are not necessarily apps that are used frequently. It could be needed to expand the investigation to Android apps as well. Second, this study could not clearly define the “purpose of use” classification. The “purpose of use” classification assumed identical functionality for apps serving multiple purposes. For example, if an app served both “eating habits” and “exercise,” the assumption was that identical functionality existed for both “purposes of use.” However, while the “D: content provision function” might be implemented for “eating habits,” it might not extend to “exercise.” Despite this, the “D: content provision function” was also assumed to be implemented in the “exercise” category for discussion purposes. This was because of its existence in the app itself. In categories such as “self-management,” “habit formation,” and “teaching materials or practice,” where the app providers did not specify distinct health care purposes, leaving them to the users, overlap between multiple “purposes of use” was assumed to be absent. Third, there are other aspects of the app’s characteristics. In this study, we investigated the characteristics of apps that could be used during a disaster in ordinary time. It is also possible that apps from genres (categories) uncovered in this study could be adapted to the health field. Finally, we believe that the results of this study are not relevant in times of immediate disaster or response to disaster but are effective in the aftermath and the recovery phase.

### Conclusions

In this study, we underscored the high utility of apps used in everyday contexts, highlighting their potential usefulness even in disaster situations owing to their reliability and reduced initial implementation.

The “D: content provision function” emerged as crucial, particularly during disasters when specific solutions are essential. Since the “D: content provision function” tended to be significantly implemented in the “sleep,” “relaxation,” and “exercise” categories, we observed the potential for health management in both the “mental” and “physical” realms even amid disasters. We believe that providing tailored content that can be used effectively during such crises is an important issue for the future.

In the future, apps in subgenres where the content provision function had a significantly high implementation rate will be studied individually to verify their effectiveness.

## Supplementary material

10.2196/56862 Multimedia Appendix 1 Categorization of the app’s purpose of use.

## Abbreviations

GEJE: Great East Japan Earthquake
