# Supplementary material for: Purposes of Smartphone Health Care Apps and the Practicality of Their Functions in Disaster Situations: Qualitative Function Assessment Study
Source: JMIR Form Res. 2025 Mar 5;9:e56862. doi: 10.2196/56862 (PMC11922814; doi:10.2196/56862)
Supplement: Multimedia Appendix 1 [file formative-v9-e56862-s001.docx]

| Purpose of use | Description |
| --- | --- |
|  |  |
| Eating habits | Applications related to eating habits, such as food management (including water) and recipes. |
| Sleep | Applications with functions related to sleep. |
| Relaxation | Applications related to meditation, relaxation, and mental health. |
| Walking | Applications with functions related to walking. |
| Running | Applications with functions related to running. |
| Exercise | Applications with functions related to workouts and training other than walking/running. Yoga and radio calisthenics also fall under this category. |
| Cycle management (menstruation/pregnancy) | Applications with menstrual and pregnancy cycle management and recording. |
| Childcare/Parenting | Applications related to children's records and growth. Includes those intended for use by parents for their children. |
| Actual service provision | Applications intended for service (with human intervention) by physical stores. |
| Self-management | Applications with no specific purpose are primarily intended for general health and physical condition management. This category also includes applications dedicated to wearable devices. |
| Habit formation | Applications that encourage habit formation, including to-do lists and task management applications. |
| Teaching materials/practice | Applications that primarily aim to provide teaching materials and methods for acquiring and practicing knowledge and methods themselves. |
| Tools for Apple Health | Applications designed to assist the IT system functionality of Apple's health care application (HealthKit). Includes applications for data integration with devices that cannot be connected as standard. |
| Not applicable | Applications that do not have healthcare-related functions. |
| Pack of applications | Multiple application products are packaged together. |
